# A Nano-Silver Loaded PVA/Keratin Hydrogel With Strong Mechanical Properties Provides Excellent Antibacterial Effect for Delayed Sternal Closure

**DOI:** 10.3389/fbioe.2021.733980

**Published:** 2021-10-08

**Authors:** Yanjun Pan, Pengfei Li, Fubang Liang, Jingyi Zhang, Jiang Yuan, Meng Yin

**Affiliations:** ^1^ Department of Cardiothoracic Surgery, Shanghai Children’s Medical Center, School of Medicine, Shanghai Jiao Tong University, Shanghai, China; ^2^ Jiangsu Key Laboratory of Biofunctional Materials, School of Chemistry and Materials Science, Nanjing Normal University, Nanjing, China

**Keywords:** delayed sternal closure, antibacterial, silver nanoparticles, keratin, poly (vinyl alcohol)

## Abstract

Delayed chest closure (DSC) is widely performed during the treatment of congenital heart diseases. However, the high prevalence of surgical site infection (SSI) in patients undergoing DSC affects prognosis negatively. Herein, we designed a suturable poly (vinyl alcohol)/keratin film loaded with silver nanoparticles (AgNPs) as an alternative material for DSC, which was named PVA/Keratin/AgNPs. The PVA/Keratin/AgNPs films exhibited significantly enhanced mechanical strength after crosslinking by sodium trimetaphosphate (STMP). These films were non-toxic, and cells proliferated with good morphology after 1 week of culture. In addition, PVA/Keratin/AgNPs films provided superior antibacterial ability, as evidenced by the eradication and lower growth rate of *Staphylococcus aureus* and *Escherichia coli*. Finally, the PVA/Keratin/AgNPs films were demonstrated to successfully cover the chest cavity temporarily and protect the chest cavity from bacterial infection. These results indicated that the PVA/Keratin/AgNPs films have great prospects to be further exploited for clinical applications in DSC.

## Introduction

Congenital heart disease (CHD) is the most common human birth defect, accounting for about a quarter of cases. For some complex CHDs (such as hypoplasia of left heart syndrome, transposition of great arteries, interruption of the aortic arch, etc.), surgical intervention is often required during the very early postnatal period, when the chest volume is small (McElhinney et al., 2000; Samir et al., 2002). Once the chest is closed immediately after surgery, it will cause a sharp increase in intrathoracic pressure occurs, leading to ventricular dysfunction and poor ventilation, eventually resulting in mortality (Nelson-McMillan et al., 2016).

The delayed sternal closure (DSC) technique, proposed in 1975, emphasizes the importance of cardiac/mediastinal mismatch after cardiac surgery and has dramatically improved the prognosis of children with unstable postoperative hemodynamics (Riahi et al., 1975). However, DSC has been reported to increase the risk of surgical site infection (SSI), mainly caused by *Staphylococcus aureus* (*S. aureus*) and *Escherichia coli* (*E. coli*). (Hansen et al., 2010). Currently, the most common method to prevent SSI after DSC is to suture sterile gloves on the skin around the incision to cover the thoracic cavity temporarily (Yabrodi et al., 2019). Although sterile gloves can avoid direct contact between external bacteria and internal tissues, their practical effects are often limited due to the lack of long-term antibacterial ability. In addition, the poor light permeability of sterile gloves makes it impossible for surgeons to deal with sudden and unexpected events in time, such as postoperative bleeding and pericardial tamponade, delaying the optimal time for treatment.

**GRAPHICAL ABSTRACT:**
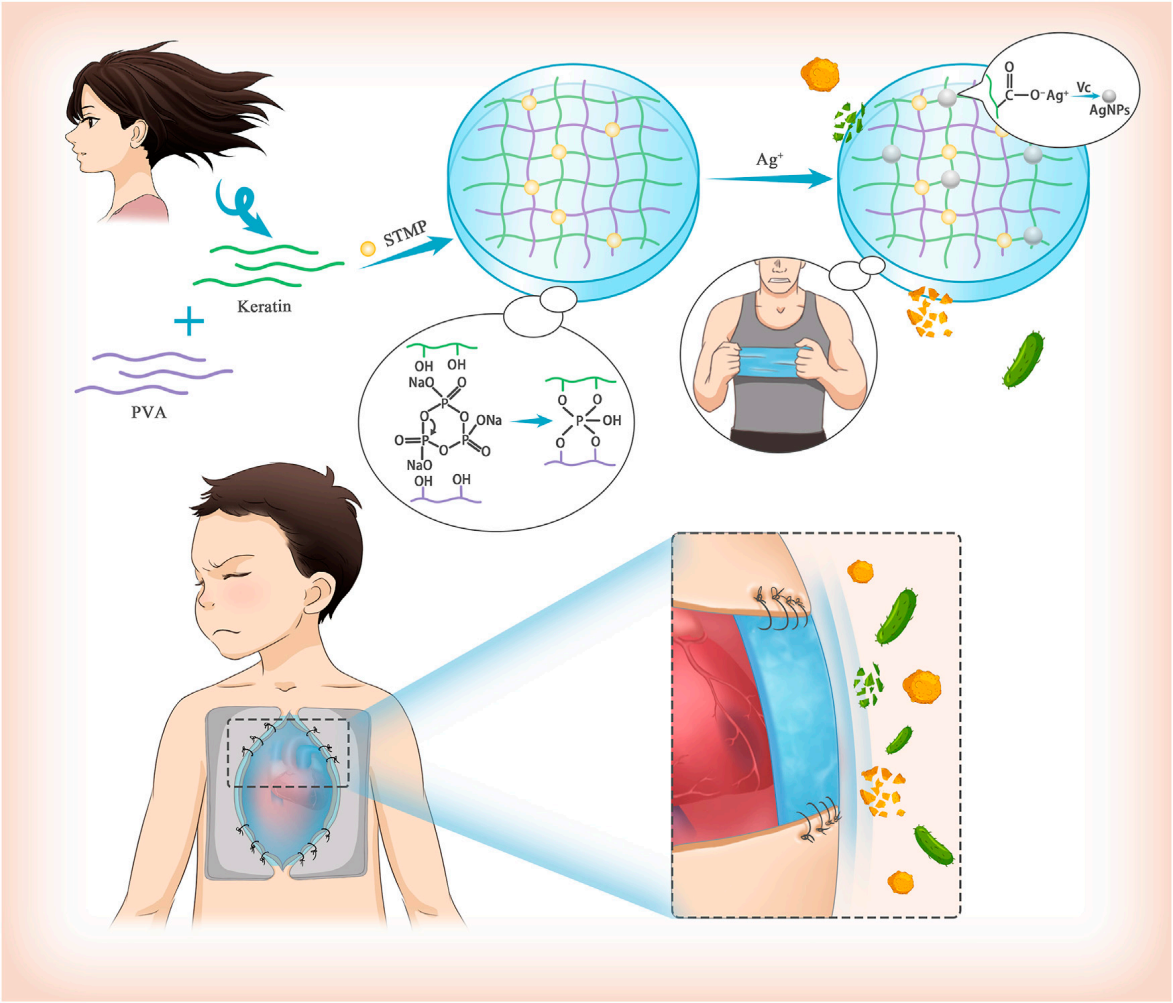


Therefore, designing a membrane-like biomaterial with good light permeability, and long-term antibacterial ability for surgical sutures may have huge prospects for clinical application in DSC(Feroz et al., 2020). Herein, we designed and prepared silver nanoparticles (AgNPs) loaded film based on poly (vinyl alcohol) (PVA) and keratin. The crosslinking between PVA and keratin by the action of sodium trimetaphosphate (STMP) provided the film with good mechanical properties to meet the requirements of surgical sutures (Leone et al., 2010; Samoila et al., 2019) while AgNPs conferred excellent antibacterial properties (Xie et al., 2017; Mabrouk et al., 2020; Fan et al., 2021). Furthermore, both *in vivo* and *in vitro* experiments were carried out.

## Materials and Methods

### Preparation of Hydrogel Films

#### Preparation of PVA Films

10 g of PVA (MW 78,000) was added to 90 ml of distilled water, followed by stirring at 90°C until PVA was completely dissolved. After cooling at room temperature until bubbles disappeared, 10 ml of PVA solution was transferred into a polytetrafluoroethylene (PTFE) plate. The plate was allowed to dry to obtain membrane-like materials and soaked in 0.12 mol/L STMP (Macklin Co. Ltd, Shanghai, China) for 24 h to complete the crosslinking reation. The films were washed with deionized water to remove unreacted crosslinkers and obtain PVA films.

#### Preparation of PVA/Keratin Films

The method used to extract keratin has been previously documented (Yuan et al., 2015; Jin et al., 2016). 2 ml keratin solution with a 100 mg/ml concentration was added to an 8 ml PVA solution. The mixed solution was stirred slowly for 2 h and then transferred to a 9 cm PTFE plate and allowed to dry in the dark. Then the films were immersed in 0.12 mol/L STMP solution 24 h for the crosslinking reaction. Finally, the films were washed with deionized water to remove unreacted crosslinker to obtain PVA/Keratin films.

#### Preparation of PVA/Keratin/AgNPs Films

The above PVA/Keratin films were immersed into 1 wt% silver nitrate solution for 2 h. After soaking and rinsing three times in distilled water, the films were immersed in 0.5 mg/ml ascorbic acid solution (Asc, AR, Sinopharm Chemical Reagent Co., Ltd, Shanghai, China) to reduce the adsorbed silver ions to AgNPs.

### Characterizations of Films

The morphology of the films was observed through scanning electron microscopy (SEM, JEOL, 6500). Attenuated total reflection-Fourier transform infrared (ATR-FTIR) spectroscopy was performed on a Bruker ALPHA II equipped with an Omni sampler over 64 scans. Surface chemical compositions were analyzed by X-ray photoelectron spectroscopy (XPS, UIVAC-PHI, Japan) using AlKα radiation. The binding energy of the C1s peaks of the carbon atoms of the hydrocarbon segments was set at 285.0 eV as the binding energy reference. Energy dispersive spectrometer (EDS) test was finished by ZEISS SIGMA HD with OXFORD X-MAS (Germany). Thermogravimetric analysis was conducted using a thermogravimetric analyzer (NETZSCH, STA449F3, Germany) from 25 to 800°C in N_2_ (10°C/min). The average diameter of nanoparticles was calculated from 100 particles by ImageJ and expressed as mean ± SD.

Meanwhile, the enzyme degradation experiment was also conducted according to the published literature (Artigas et al., 1981). Briefly, the PVA and PVA/Keratin/AgNPs films were immersed into phosphate-buffered saline (PBS) with tryspin (2 μg/L) at 37°C. At a pre-determined interval, the films were taken out to record the weight after 2 h of drying. The degradation rate (D%) was calculated according to the formula:

$$\text{D\%}=\frac{\left({\text{m}}_{\text{0}}-{\text{m}}_{\text{t}}\right)}{{\text{m}}_{\text{0}}}\text{× }100\text{\%}$$ (1)

where m_0_ is the initial weight of the films, m_t_ is the weight of films at certain time.

### Mechanical Properties of Films

A universal tensile tester machine was used to perform a tensile test at room temperature, with an extension rate of 50 mm/min. Briefly, strip-shaped hydrogels (10 mm × 40 mm) were first prepared, and then the thickness of each sample was measured using a spiral micrometer. According to the obtained stress-strain curve, the initial elastic modulus (the slope of the initial linear region of the stress-strain curve), the elongation at break, and the maximum tensile strength were calculated.

An Electro-Force 3200 testing machine (TA, United States) was used to evaluate the dynamic mechanical properties of the films. The loading and unloading force of PVA films was set between 0.01 and 0.1 N and between 0.1 and 1 N for PVA/Keratin and PVA/Keratin/AgNPs films. Then, different films were stretched dynamically in a salt bath. The shape recovery rate (R%) of each cycle was calculated according to the formula:

$$\text{R\%}=\left({\text{ε}}_{\text{x}}-{\text{ε}}_{\text{y}}\right)/\left({\text{ε}}_{\text{x}}-{\text{ε}}_{\text{z}}\right)\times 100\text{\%}$$ (2)

(Ahn and Kasi, 2011; He et al., 2017).Where ε_x_, ε_y_, and ε_z_ represent the strain when the maximum stress was loaded, the strain when the stress was fully unloaded, and then strain before the stress was loaded in each cycle, respectively.

At least three parallel samples were tested.

### Cell Compatibility of Films *in Vitro*

Human smooth muscle cells (HSMCs) were cultured on different media in Dulbecco’s modified eagle’s medium (DMEM/High Glucose, Hyclone, United States) with 10% Fetal Bovine Serum (FBS, Biological Industries, Israel) and 1% antibiotic–antimycotic at an atmosphere of 37°C, 5% CO_2_, and 95% humidity. Blank coverslips were used for the control. Briefly, the films were sterilized with anhydrous alcohol for 24 h and then immersed in sterile PBS solution thoroughly and rinsed for a total of 3 times. HSMCs were seeded at a density of 1.0×10^4^ cells/well, and the medium was changed every other day. A Cell Counting Kit-8 (CCK-8) (Dojindo Lab., Japan) was used to evaluate the proliferation of HSMCs. The detection time was set at 1, 4, and 7 days. Moreover, Rhodamine phalloidin (Thermo Fisher, United States) staining was used to observe the morphology of HSMCs after 4 days of cell culture. HSMCs were provided by the Shanghai Academy of Life Science Cell Bank and the Chinese Academy of Science (Shanghai, China).

### Antibacterial Performance of Films *in vitro*

#### The Antibacterial Rate of Different Films

The bacterial solutions of *S. aureus* and *E. coli* were diluted with phosphate buffered saline (PBS) buffer (pH 7.4) to a concentration of 1 × 106 CFU/ml. Different films were put in a 24-well plate, and 1 ml of the diluted bacterial solution was added. The plate was placed in a bacteria incubator to co-cultivate for 24 h. Then the culture medium was aspirated and diluted to an appropriate concentration with PBS buffer. 100 μL of the diluted solution was taken to inoculate on the bacterial nutrient agar plate, and it was smeared evenly with a bacterial smear stick. The bacterial agar plate was put upside down in a bacterial incubator for 24 h. A digital camera was used to photograph the growth of the colonies, and the number of colonies on the bacterial agar plate was counted. Finally, the antibacterial rate of different materials was calculated by the following formula:

$$\text{Antibacterial}\;\text{rate}\left(\text{\%}\right)=\left[\frac{\left(\text{C}-\text{M}\right)}{\text{C}}\right]\times 100\text{\%}$$ (3)

(Sun et al., 2020).where C is the average number of colonies on the bacterial agar plate of the control group, and M is the average number of colonies of a different film group.

#### Morphologies of Bacteria

The bacterial solution of each group was taken out and centrifugated, and the supernatant was removed. 1 ml of 2.5% glutaraldehyde solution was added to fix the bacteria for 2 h. The solution was centrifuged once more, followed by removing the supernatant. 30, 50, 75, 85, 90, 100% ethanol was used for gradient dehydration successively for 10 min each time. After the dehydration was complete, the unremoved ethanol was replaced with isoamyl acetate. Finally, 10 μL of the bacterial solution was dropped on the copper sheet, freeze-dried, and gold sprayed. The morphology of the bacteria was observed using SEM (S-4800).

#### Bacterial Kinetics

The bacteria solution was diluted to a concentration of 10^5^ CFU/ml with PBS buffer, followed by adding 500 μL of bacterial solution and 500 μL of culture medium to a 24-well plate. Subsequently, the film was cut to a suitable size and put into each well, and no samples were added to the blank control group. The plate was put into a 37°C constant temperature incubator for 24 h. Samples of the bacterial solution were taken at 3, 6, 12, and 24 h after culture. A microplate reader was used to detect the absorbance value of the bacterial solution at optical density (OD) 600 nm to plot the antibacterial kinetics curve.

### Application of Films for Delayed Sternal Closure

All procedures were conducted respecting the ARRIVE guidelines and approved by the Animal Ethics Committees of Shanghai Children’s Medical Center, Shanghai Jiaotong University. Furthermore, the experiments strictly followed the National Institutes of Health guide for the care and use of laboratory animals (NIH Publications No. 8023, revised 1978). A total of six New Zealand white rabbits underwent delayed sternal closure surgery. First, sodium pentobarbital with a concentration of 3% (1 ml/kg) was slowly injected via the ear-marginal vein to induce anesthesia. Then, the rabbits were endotracheally intubated, and 0.5% isoflurane was used to maintain anesthesia. The ventilation frequency was set at 18 times/minute. Next, the chest skin of the rabbits was prepared and disinfected with iodophor, followed by establishing a midline incision. Different hydrogel membranes were used for wound closure, and the rabbits in the control group were sealed with sterile surgical gloves. Equal amounts of *S. aureus* and *E. coli* bacteria were seeded with cotton swabs at the junction between the material and tissue. 3, 6, 9, and 12 h after surgery, bacteria were collected and cultured for 24 h. Then the absorbance at a wavelength of 600 nm was measured. Twelve hours after the operation, bacteria were inoculated on a bacterial nutrient agar plate to observe colony formation.

### Data Analysis

All data were obtained from at least three parallel samples and expressed as mean ± SD. GraphPad Prism 8.0 Software (Graphpad Software Inc, La Jolla, CA) was used for analyses, and One-way ANOVA was used to evaluate the statistical differences between groups. *p* < 0.05 was considered statistically significant.

## Results

### Characterizations of Films

**FIGURE 1:**
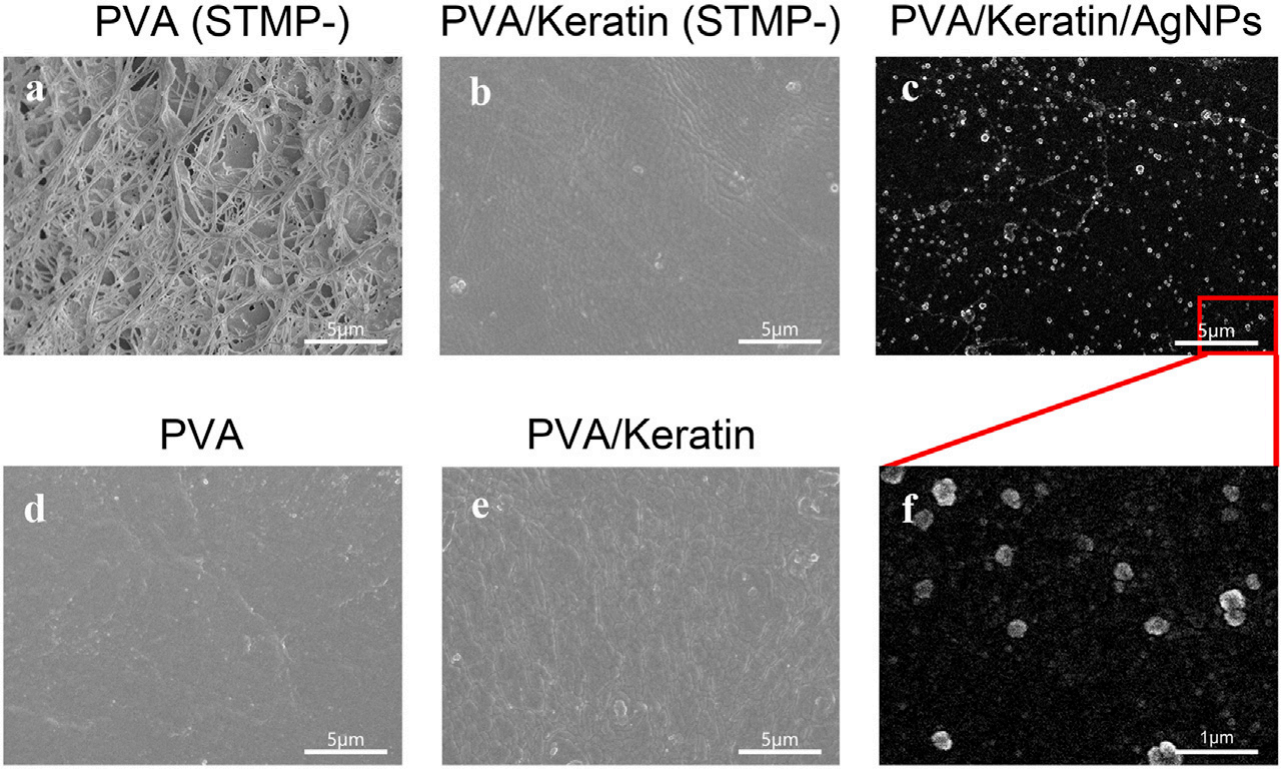
Microstructure of **(A)** PVA without crosslinking by STMP, **(B)** PVA/Keratin without crosslinking by STMP, (**C)** PVA/Keratin/AgNPs at bar = 5 μm, **(D)** PVA after crosslinking by STMP, **(E)** PVA/Keratin after crosslinking by STMP, **(F)** PVA/Keratin/AgNPs at bar = 1 μm.

As shown in Figure 1A, the morphology of the PVA film before crosslinking reaction consisted of fibrous and loose structures, while the STMP treated PVA film exhibited a smooth and compact surface without holes (Figure 1B). Interestingly, the PVA/Keratin hydrogel before STMP treatment displayed a flat surface (Figure 1C), similar to PVA and PVA/Keratin hydrogels crosslinked by STMP (Figure 1D). This result may be due to the formation of hydrogen bonds between PVA and keratin during the evaporation of deionized water. The SEM images of PVA/Keratin/AgNPs films revealed that AgNPs were successfully fixed and exhibited spherical shapes without aggregation (Figures 1E,F).

**FIGURE 2:**
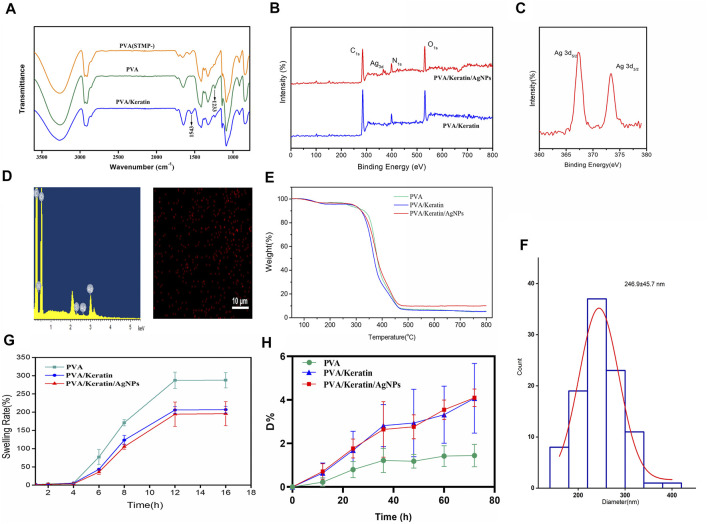
Characterizations of hydrogels. **(A)** ATR-FTIR diagram of PVA, PVA treated with STMP and PVA/Keratin. **(B)** The wide scan XPS spectra of PVA/Keratin and PVA/Keratin/AgNPs. **(C)** Ag3d high-resolution spectra of PVA/Keratin/AgNPs. **(D)** EDS spectrum of PVA/Keratin/AgNPs and SEM-EDX elemental mapping image of Ag. **(E)** Thermogravimetric curves of PVA, PVA/Keratin, and PVA/Keratin/AgNPs. **(F)** Diameter distribution of silver nanoparticles. **(G)** Swelling rate of PVA, PVA/Keratin, and PVA/Keratin/AgNPs. **(H)** Enzymatic degradation of PVA, PVA/Keratin, and PVA/Keratin/AgNPs.

Furthermore, a new absorption peak at 1,233 cm^−1^ in the STMP treated PVA and PVA/Keratin spectra represented the pyrophosphate produced by the reaction of PVA with STMP. In addition, for the PVA/Keratin composite film, the absorption peak at 1,543 cm^−1^ could be the human hair keratin amide II band of protein, indicating the successful preparation of PVA/Keratin (Figure 2A). The chemical composition change of films was analyzed using the XPS spectra (Figures 2B,C). The prominent peaks at 370 and 400 eV on PVA/Keratin/AgNPs spectra were attributed to silver and nitrogen. A double peak at 367.4 eV (3d_5/2_) and 373.4 eV (3d_3/2_) with 6.0 eV separation were observed in high-resolution silver spectra, indicating that the formed silver nanoparticles were zero-valent.

In addition, there is strong evidence for Ag signal that originates mainly from nanoparticles, and elemental mapping of Ag also revealed that the Ag nanoparticles formation (Figure 2D). These results suggested that silver nanoparticles were successfully immobilized *in situ* on the PVA/Keratin surface using ascorbic acid, and most silver nanoparticles had diameters between 200 and 300 nm (Figure 2F). The thermal decomposition curves of PVA/Keratin/AgNPs film under the protection of N_2_ was shown in Figure 2E. The initial weight loss of PVA/Keratin/AgNPs blew 200°C is attributed to the evaporation of residual moisture. The second stage starting from 260 to 480°C and the weight loss was approximately 70%, corresponds to the degradation of keratin and PVA. At the temperature of 480°C, PVA/Keratin/AgNPs had a smaller weight loss (89.9 wt%) compared to that of PVA/Keratin (92.35 wt%) or PVA/Keratin (92.35 wt%). This may be attributed to that the Ag acts as an interchain cross-linker to match two carboxyl groups of two neighboring protein chains, and then increased the thermal stability of PVA/Keratin/AgNPs film. Weight loss was not observed after increasing the temperature in the range of 480–800°C, the residual weight difference of PVA/Keratin/AgNPs and PVA/Keratin indicated the content of the AgNPs was 2.33 wt%. By the way, PVA/Keratin/AgNPs film was performed for DSC purposes that do not need a higher temperature.

The swelling rate of PVA/Keratin and PVA/Keratin/AgNPs films was lower than PVA (Figure 2G). Besides, as shown in Figure 2H, the degradation rate of PVA only reached 1.44% after 3 days of incubation in trypsin, while the degradation rates of PVA-Keratin and PVA/Keratin/AgNPs films increased to about 4% with no significant difference, which may attribute to the component of keratin could be degraded by the trypsin. All the degradation rate was less than 5%, suggested that only slightly degradation of the films would occur during delayed sternal closure.

### Mechanical Properties of Films

**FIGURE 3:**
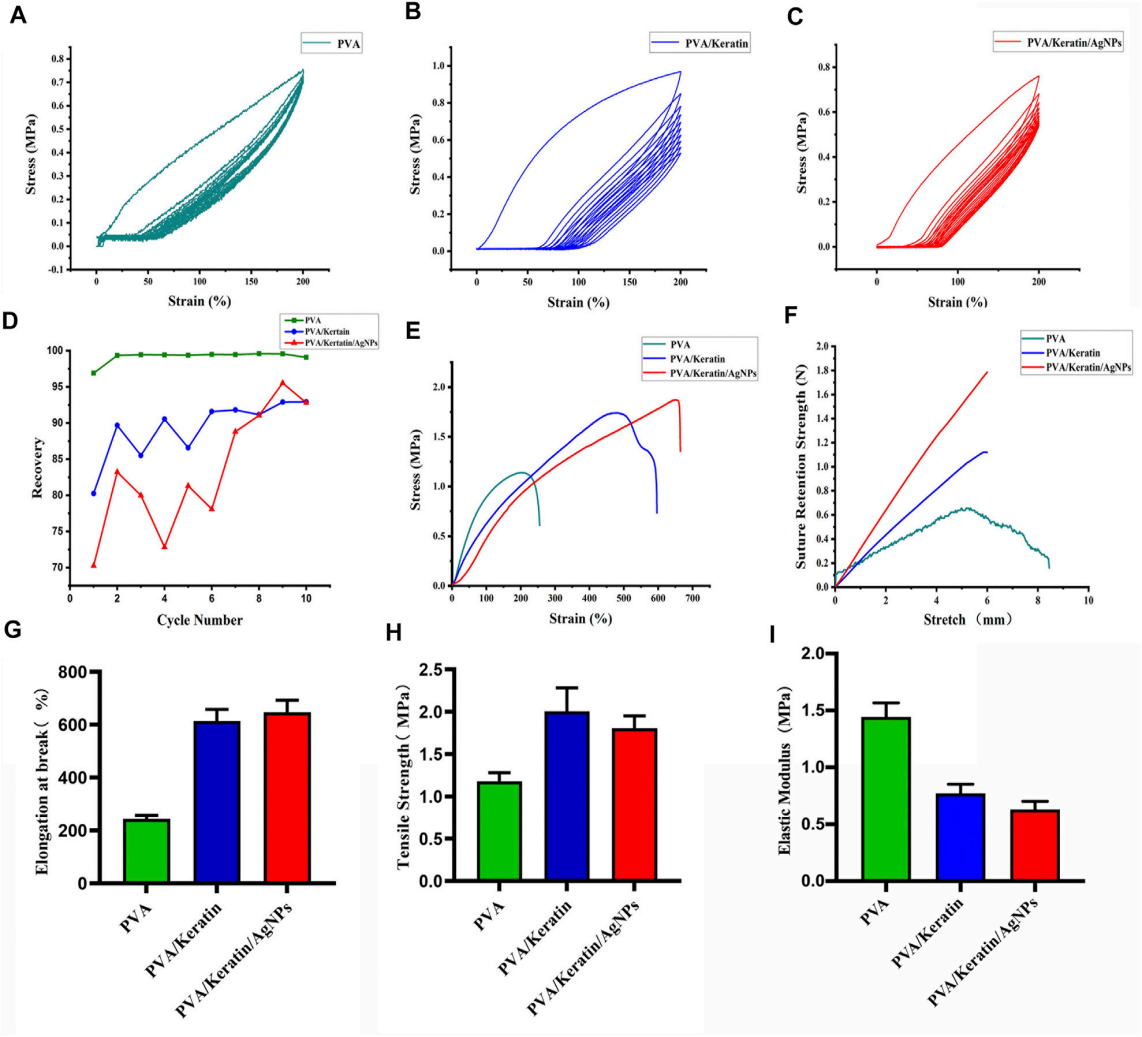
Mechanical properties of films. **(A–C)** Dynamic stretching test of PVA, PVA/Keratin, and PVA/Keratin/AgNPs. **(D)** The R% of each cycle for each film. **(E)** Tensile strain-stress curves of films. **(F)** Suture retention strength of films. **(G–I)** Elongation at break, tensile strength, and Elastic modulus of films.

The dynamic mechanical behaviors of different films were analyzed through the rheometer and the dynamic mechanical extensometer. The stress-strain hysteresis between tension and recovery of the three materials during the dynamic stretching test is shown in Figures 3A–C. The R% was calculated by the formula was shown in Figure 3D. The R% of PVA was close to 1, and both PVA/Keratin and PVA/Keratin/AgNPs were less than 1.

The tensile test showed that pure PVA presented with insufficient mechanical strength (Figure 3E). After crosslinking with keratin, the mechanical strength was reinforced, and further enhanced with nanoparticle loading, suggesting that PVA/Keratin/AgNPs films could withstand greater tensile strength. The suture retention strength was also evaluated (Figure 3F). Both PVA/Keratin and PVA/Keratin/AgNPs films did not break within the test range. However, PVA films exhibited almost no suture retention strength and were easily broken. The elongation at break (Figure 3G), tensile strength (Figure 3H), and elastic modulus (Figure 3I) were also calculated. Results showed that PVA/Keratin and PVA/Keratin/AgNPs were not easily deformed.

### Cell Compatibility of Films

**FIGURE 4:**
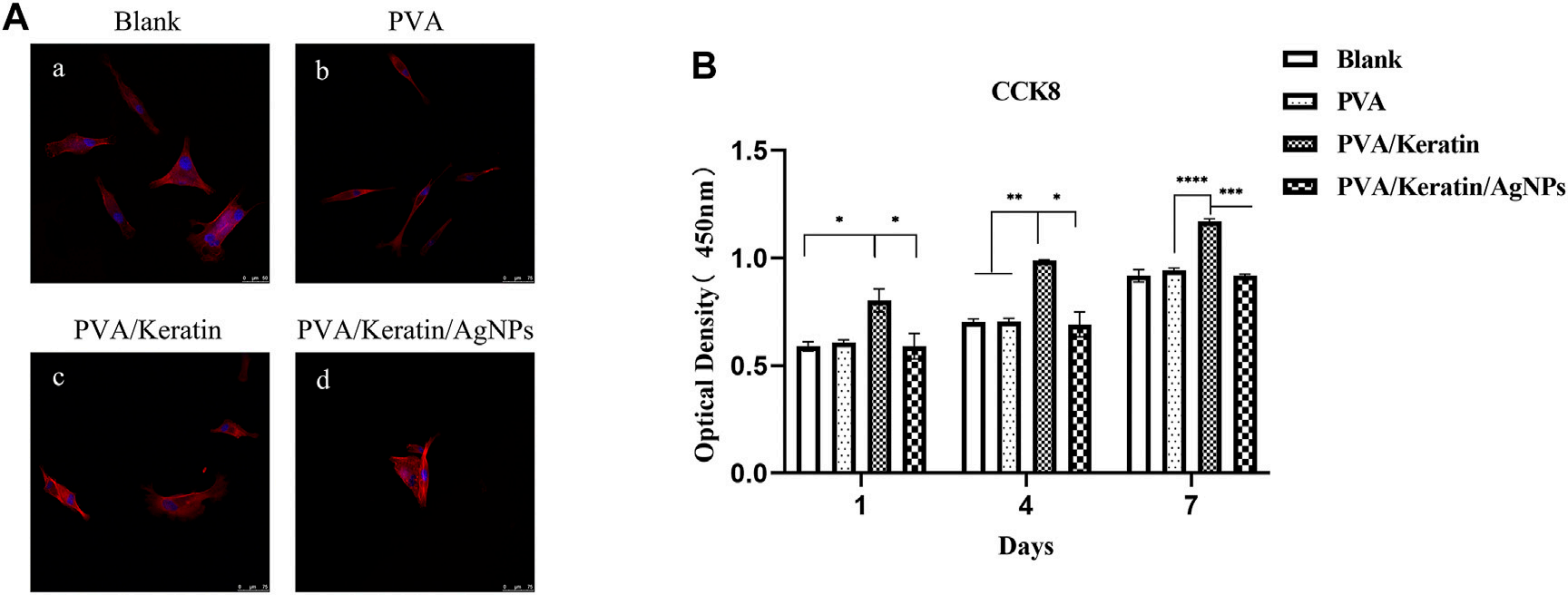
HSMCs morphology and proliferation on different materials. **(A)** Rhodamine staining photomicrograph of HSMCs (bar = 75 μm). **(B)** The optical density within 1 week of culturing (“*” represents *p* < 0.05).

As shown in Figure 4A, HSMCs were spread on PVA/Keratin/AgNPs films, indicating that PVA/Keratin/AgNPs films were non-toxic. Besides, after 1 week of culture, there was a statistically significant difference in cell proliferation between the PVA/Keratin and other groups (Figure 4B). Cells proliferated faster on PVA/Keratin films, which might be attributed to the effect of keratin. Although cell proliferation in the PVA/Keratin/AgNPs group was lower than that in the PVA/Keratin group, there was no significant statistical difference between the PVA/Keratin/AgNPs and the control group, showing that the PVA/Keratin/AgNPs films did not exhibit an inhibitory effect on cells.

### Evaluation of Antibacterial Ability *in Vitro*

**FIGURE 5:**
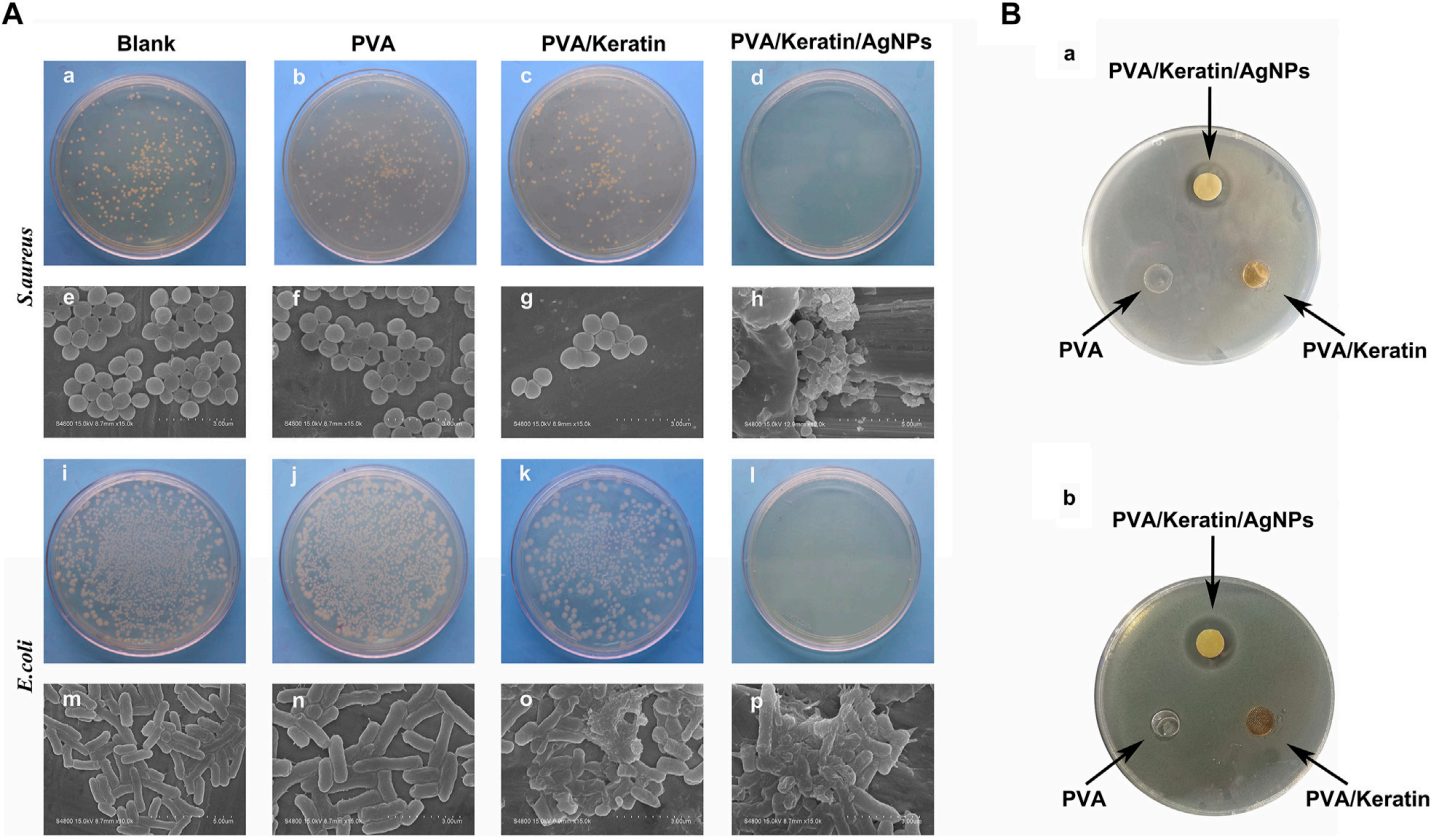
Improved antibacterial performance of PVA/Keratin/AgNPs. **(A)** Photographs of bacterial colonies formed by *S. aureus* (a–d) and *E. coli* (i–l) after 24 h of co-culturing with different materials, and the representative SEM images of *S. aureus* (e-h) and *E. coli* (m–p). **(B)** Inhibition zone of different materials to *S. aureus* (a) and *E. coli*.

**FIGURE 6:**
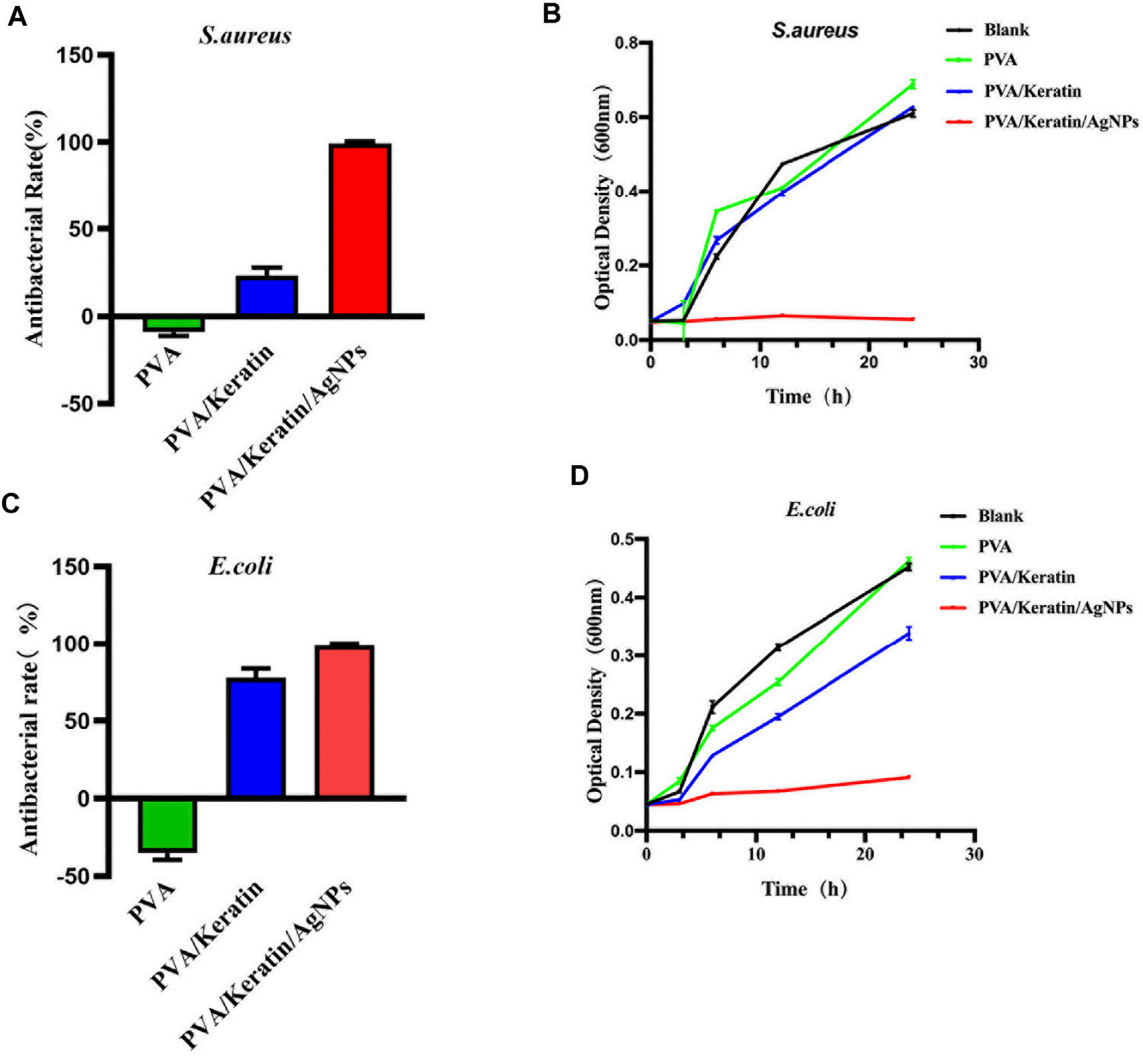
Antibacterial rate and bacterial kinetics of *S. aureus* and *E.Coli in vitro*. **(A, C)** Antibacterial rate of different materials against *S. aureus* and *E. Coli*. **(B, D)** Bacterial kinetics of *S. aureus* and *E. Coli* on different materials.

After co-cultivating the bacterial liquid with different film samples, the antibacterial activity testing substantiated a significant antibacterial activity against *S. aureus* and *E. coli* exerted by PVA/Keratin/AgNPs*.* Compared with other groups, fewer bacterial colonies were observed in PVA/Keratin/AgNPs (Figures 5Aa–D,i–l), and both bacteria were eradicated (Figures 5Ah,p). Besides, during the inhibition zone experiment, an apparent bacteria-free area was observed around the PVA/Keratin/AgNPs (Figure 5B), suggesting that PVA/Keratin/AgNPs film could achieve antibacterial function through the release of AgNPs and kill any bacteria around the material by direct contact. For further validation, the antibacterial rates of different materials were calculated. The results showed that the PVA/Keratin/AgNPs film exhibited the best antibacterial rate (Figures 6A,C), as evidenced by bacterial kinetics (Figures 6B,D).

### Evaluation of Antibacterial Ability *in Vivo*

**FIGURE 7:**
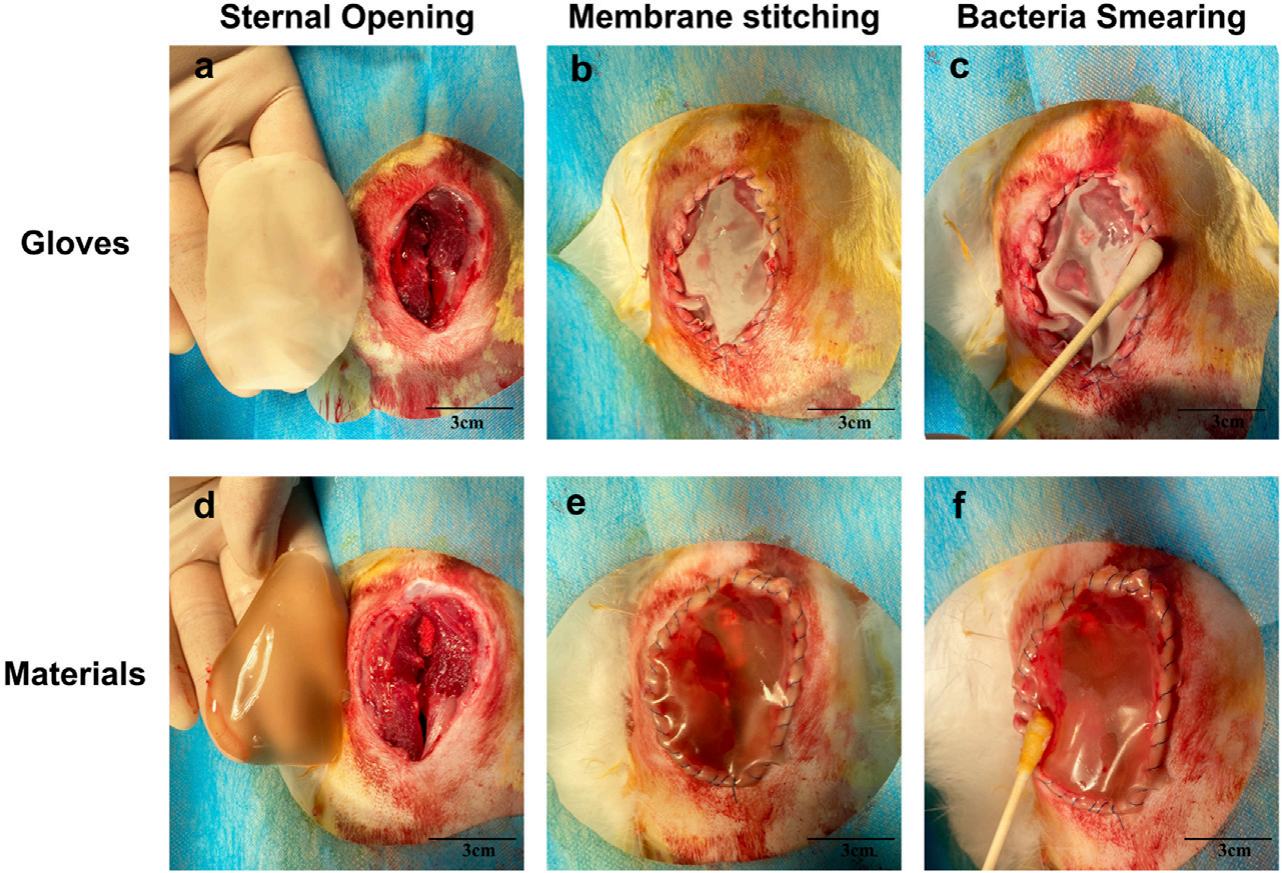
**(A–C)** Performing DSC with surgical glove sheet, and **(D–F)** with materials.

**FIGURE 8:**
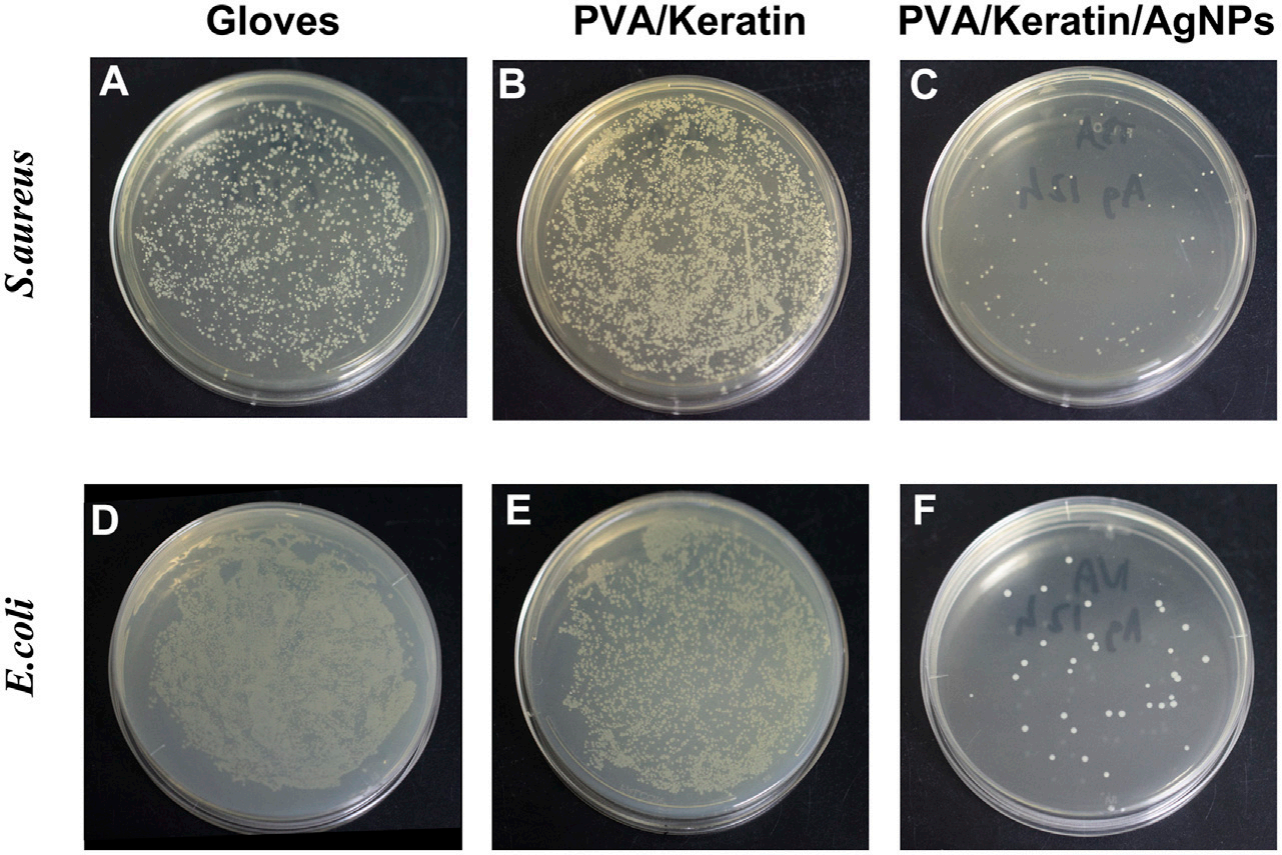
Photographs of bacterial colonies formed by **(A–C)** *S. aureus* and **(D–F)** *E. coli* of different materials after 24 h of culturing.

**FIGURE 9:**
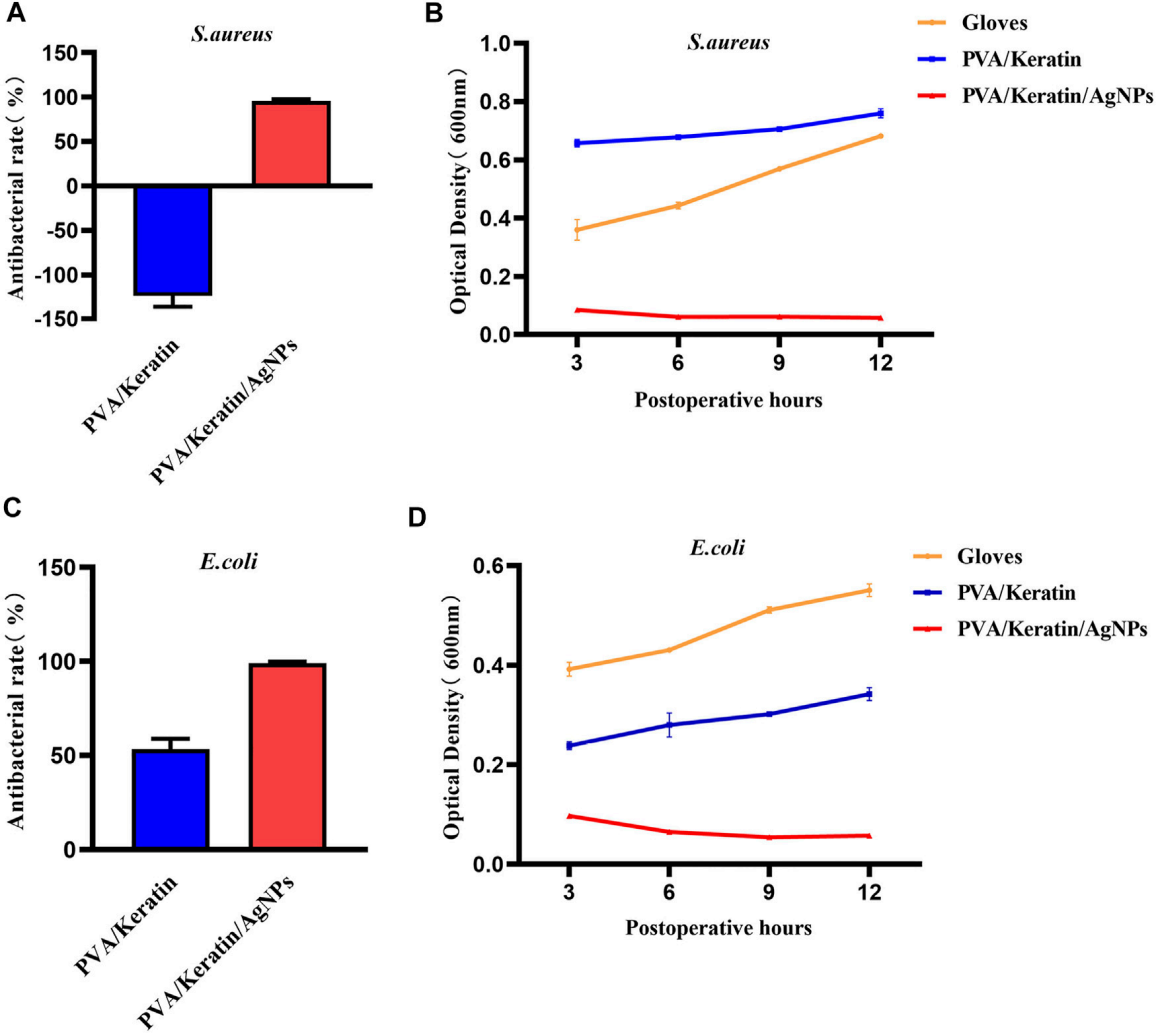
Antibacterial rate and bacterial kinetics of *S. aureus* and *E.Coli in vivo*. **(A, C)** Antibacterial rate of different materials against *S. aureus* and *E. Coli*. **(B, D)** Bacterial kinetics of *S. aureus* and *E.Coli* on different materials.

As shown in Figure 7, the PVA/Keratin/AgNPs film possessed sufficient toughness and could be tightly sutured to the incision to close the chest cavity, similar to surgical glove sheet. However, compared with a surgical glove sheet, PVA/Keratin/AgNPs film had the advantage of light transmittance, making it possible for doctors to have a direct view of the thoracic cavity at any time. Meanwhile, the results of the formation of bacterial colonies (Figure 8), the antibacterial rate (Figures 9A,C) and bacterial kinetics (Figures 9B,D) proved that the PVA/Keratin/AgNPs film had excellent antibacterial ability in practice. It is worth noting that the *E. coli* proliferated less on PVA/Keratin film compared to glove sheet, indicating that PVA/Keratin seemed to have a certain degree of antibacterial effect on *E. coli*, consistent with the results *in vitro*.

## Discussion

A high incidence of postoperative tamponade condition after open-heart surgery has been reported, especially in pediatric patients due to compromised cardiac and pulmonary function and large heart/thoracic volume ratio (Riahi et al., 1975; Gangahar et al., 1981). Leaving the sternum open can lead to a significant increase in blood pressure and decrease central venous pressure which can optimize patient hemodynamics and minimize complications associated with low output syndrome (Shore et al., 1982). Accordingly, DSC has become a widely used technique after congenital heart surgeries in children and has dramatically improved their survival rate (Hakimi et al., 1994; Alexi-Meskishvili et al., 1995). A study by McElhinney et al. found that 39% of neonates and 22% of infants that underwent cardiac surgery required DSC (McElhinney et al., 2000). However, DSC has been reported to prolong skin healing, increasing the incidence of SSI(Badia et al., 2017; Ban et al., 2017). According to an analysis of the Society of Thoracic surgeons Congenital Heart Surgery Database, DSC-related infections in infants or newborns was 18.7%, compared to only 6.6% in patients without DSC (*p* < 0.001). When a more restrictive criterion was applied, a statistically significant difference was found between the SSI incidence in DSC patients and those without DSC (9.8 vs. 3.8%, *p* < 0.001. Furthermore, the increased risk of SSI-related mortality ranged from 2 to 11 times, and nearly 75% of postoperative deaths were related to SSI(Nelson-McMillan et al., 2016). Clinically, surgeons often use surgical glove sheets to temporarily close the incision and prevent direct contact between external bacteria and tissues in the chest cavity. However, DSC usually lasts for 72 h, leading to a limited effect of sterile gloves due to the lack of long-term antibacterial mechanism. Therefore, there is an urgent need for more research and development of a functional barrier membrane that can be used for DSC.

Given that antibiotic abuse has promoted bacterial mutation and the development of drug resistance, a new type of antibacterial dressing with silver ions has attracted the attention of researchers. Since Moyer et al. firstly used 0.5% silver nitrate and 1% silver sulfadiazine to treat burn wounds in the 1960s (Klasen, 2000), the good antibacterial properties of silver ions have been studied extensively and are already widely implemented in the medical field (Barillo and Marx, 2014; Xie et al., 2017). Nonetheless, as heavy metal ions, excessive silver ions can cause toxicities involving different organs, including the skin, eyes, and the respiratory, hepatobiliary, nervous, and reproductive systems (Gaiser et al., 2013; Gao et al., 2017).

The silver monomers of AgNPs and their composite materials have been reported to have strong antibacterial properties against microorganisms (Xiu et al., 2012). Moreover, the antibacterial effect of AgNPs has been reported to be increased when particle diameter was decreased (Raza et al., 2016). In general, silver nanoparticles are biologically safer and exhibit less toxicity than silver ions. It has been documented that nanoparticle-containing dressings did not produce systemic or local toxicity when applied to non-burn skin wounds (Yamanaka et al., 2005; Marin et al., 2015; Fan et al., 2021). Furthermore, the AgNPs loaded on the carrier can be released continuously and maintain a relatively constant concentration, achieving the purpose of long-lasting antibacterial activity (Li et al., 2010). Studies have shown that AgNPs can kill bacteria by affecting the living environment of bacteria, destroying cell walls, inhibiting DNA replication or enzyme activation. AgNPs can also effectively inhibit various pathogenic Gram-positive and Gram-negative bacteria (Xu et al., 2020), hence providing a new means towards fighting multidrug resistance (Thomas et al., 2017). After loading AgNPs, the PVA/Keratin/AgNPs hydrogels we obtained exhibited excellent antibacterial ability, *in vitro* and *in vivo* experiments demonstrated that the proliferation of both Gram-negative and Gram-positive bacteria was inhibited.

Interestingly, inconsistent findings were obtained regarding the inhibition of Gram-negative and Gram-positive bacteria by the PVA/Keratin hydrogel. The PVA/Keratin hydrogel exerted a certain inhibitory effect on Gram-negative bacteria compared to the control group. This observation could be explained by differences in the structure of Gram-negative and Gram-positive bacteria, which could lead to their different reactions to materials (Xu et al., 2020) or the presence of a large number of amino groups in keratin, which may form a structure similar to aminoglycoside antibiotics during the crosslinking process (Sadeghi et al., 2020). Further studies are essential to better understand the differences in inhibition efficacy of Gram-negative and Gram-positive bacteria.

Moreover, for DSC application, the barrier material used should meet the requirements of the light transmittance for a direct view of the chest cavity and mechanical strength for sutures. Based on this, PVA and keratin were introduced to prepare a composite film with good light transmittance (Shavandi et al., 2017), and the PVA/Keratin and PVA/Keratin/AgNPs films also possessed strong mechanical strength. It is widely acknowledged that the R% of an ideal elastic material is 1, while the R% of a viscoelastic material is much less than 1. The present study proved that the keratin crosslinked films underwent a significant transition from elasticity to viscoelasticity, and the toughened films exhibited a time dependent response. This performance is very welcome to surgeons, indicating that it could be a potential skin-simulating material for DSC.

## Conclusion

In this study, the nano-silver loaded poly (vinyl alcohol)/keratin (PVA/Keratin/AgNPs) hydrogels exhibited good light-permeability, mechanical strength, and antibacterial properties. These unique features enabled a direct view of the thoracic cavity during DSC and concomitantly protected internal organs from bacterial infection. Accordingly, PVA/Keratin/AgNPs films have huge prospects for clinical application in delayed sternal closure.

## Limitation

In the present study, the antibacterial function of the PVA/Keratin/AgNPs film was evaluated for a short period. A longer observation time is necessary to substantiate our findings. In the meantime, inconsistent results were obtained for Gram-negative and Gram-positive bacteria inhibition by the PVA/Keratin hydrogel. More studies are required to ascertain the effects of the PVA/Keratin hydrogel on Gram-negative bacterial inhibition.

## Data Availability

The original contributions presented in the study are included in the article/supplementary material, further inquiries can be directed to the corresponding authors.

## Abbreviations

AgNPs, silver nanoparticles; ATR-FTIR, attenuated total reflection-fourier transform infrared spectroscopy; CHD, congenital heart disease; CCK-8, cell counting Kit-8; D%, degradation rate; DSC, delayed sternal closure; *E. coli*, *Escherichia coli*; HSMCs, human smooth muscle cells; OD, optical density; PBS, phosphate buffered saline; PTFE, polytetrafluoroethylene; PVA, poly (vinyl alcohol); R%, shape recovery rate; *S. aureus*, *staphylococcus aureus*; STMP, trimetaphosphate; SEM, scanning electron microscopy; XPS, X-ray photoelectron spectroscopy.
